# Supplementary material for: The Value of Zero-filling in In Vivo MRS
Source: Magn Reson Imaging. Author manuscript; Available in PMC 2023 Mar 15. (PMC10015433; doi:10.1016/j.mri.2022.07.011)
Supplement: 1 [file NIHMS1879459-supplement-1.pdf]

Supplementary material:

Full Matlab code to repeat the calculation performed and produce Figure 1

```
%The purpose of this script is to test the idea that zero-filling retrieves
%information from the imaginary spectrum in the case of quadrature
%detection, real spectrum modeling
%How many times am I repeating this simulation to assess errors?
n_offsets=10000;
%simulate time-domain data
time = repmat((0:1:1023).'/1000,[1 n_offsets]); %in s
offset=repmat(-500+rand(1,n_offsets)*1000,[1024 1]); %in Hz
%Set up a matrix of time-domain data that has 200 ms T2.
T2=0.1;
data = exp(1i*offset.*time).*exp(-1*time/T2);
%Inject in amplitudes that are variable
amplitudes=0.8+rand(1,size(data,2))*0.4;
for ii=1:size(data,2)
    data(:,ii)=data(:,ii)*amplitudes(ii);
end
```

bioRxiv preprint doi: https://doi.org/10.1101/2022.05.31.494144; this version posted June 1, 2022. The copyright holder for this preprint (which was not certified by peer review) is the author/funder, who has granted bioRxiv a license to display the preprint in perpetuity. It is made available under aCC-BY 4.0 International license.

```
%set the zero-filling factor
ZF=2;
%NOISE BLOCK
noise_amplitude=0.3;
data = data+noise_amplitude*(randn(1024, n_offsets)+1i*randn(1024, n_offsets));
%END NOISE BLOCK (comment out for noise-free 'data')

% Fourier transform the time-domain data with zero-filling factor ZF

spectra_nozf = fftshift(fft(data, 1024,1),1);
spectra_zf = fftshift(fft(data, 1024*ZF,1),1);
%Model the real part of the spectrum as an Absorption-mode Lorentzian
%signal (should be the exact model ignoring noise)
freq_nozf=linspace(-500,500,1024);
freq_zf=linspace(-500,500,1024*ZF);
%manually plot the data and the model to try to figure out reasonable
%starting values for the width and
%plot(freq,spectra,'k')
%hold on
%plot(freq,LorentzianModel([200 -1 0],freq),'r')
%hold off
%starting values (chosen without great care, but ballpark)
init=[200 -1 0]; %I suspect we will have to pick the offset starting value to be closer.
%Quick scan of the spectrum to find the max
[peaks ind_nozf]=max(real(spectra_nozf),[],1);
[peaks ind_zf]=max(real(spectra_zf),[],1);
%starting values (chosen without great care, but ballpark)
outputs = zeros([n_offsets 3]);
nlinopts = statset('nlinfit');
nlinopts = statset(nlinopts,'MaxIter',400,'TolX',1e-6,'TolFun',1e-6,'FunValCheck','off');
%modelFun = @(x,freq) LorentzianModel(x,freq);
for ii=1:size(data,2)
init(3)=freq_nozf(ind_nozf(ii));
   outputs_nozf(ii,:) = nlinfit(freq_nozf, real(spectra_nozf(:,ii)).' , @(x,freq) LorentzianModel(x,freq), init, nlinopts);
% add weights to the data
init(3)=freq_zf(ind_zf(ii));
   outputs_zf(ii,:) = nlinfit(freq_zf, real(spectra_zf(:,ii)).' , @(x,freq) LorentzianModel(x,freq), init, nlinopts); % add
weights to the data
end
subplot(1,3,1)
plot(freq_nozf,spectra_nozf(:,10),'o',freq_nozf,LorentzianModel([outputs_nozf(10,:)],freq_nozf),'r')
set(gca,'YTick',[]);
set(gca,'Box','off');
subplot(1,3,2)
plot(amplitudes,outputs_nozf(:,1)/199.3435*2,'.k',amplitudes,outputs_zf(:,1)/199.3435*2,'.r')
set(gca,'Box','off');
%For this linewidth the amplitude is 199.3435; noiseless fitting CV is 0.1%
%limited by modeling options nlinopts
Error_zf=(amplitudes.'-outputs_zf(:,1)/199.3435*2)./amplitudes.';
Error_nozf=(amplitudes.'-outputs_nozf(:,1)/199.3435*2)./amplitudes.';
CV_model_error_nozf=std(Error_nozf)
CV_model_error_zf2=std(Error_zf)

%Additional section added to check there is no additional value in more
%zero-filling
```

bioRxiv preprint doi: https://doi.org/10.1101/2022.05.31.494144; this version posted June 1, 2022. The copyright holder for this preprint (which was not certified by peer review) is the author/funder, who has granted bioRxiv a license to display the preprint in perpetuity. It is made available under aCC-BY 4.0 International license.

```
%set the zero-filling factor
ZF=4;
%END NOISE BLOCK (comment out for noise-free 'data')
% Fourier transform the time-domain data with zero-filling factor ZF
spectra_zf = fftshift(fft(data, 1024*ZF,1),1);
%Model the real part of the spectrum as an Absorption-mode Lorentzian
%signal (should be the exact model ignoring noise)
freq_zf=linspace(-500,500,1024*ZF);
%starting values (chosen without great care, but ballpark)
init=[200 -1 0]; %I suspect we will have to pick the offset starting value to be closer.
%Quick scan of the spectrum to find the max
[peaks ind_zf]=max(real(spectra_zf),[],1);
%starting values (chosen without great care, but ballpark)
outputs = zeros([n_offsets 3]);
nlinopts = statset('nlinfit');
nlinopts = statset(nlinopts,'MaxIter',400,'TolX',1e-6,'TolFun',1e-6,'FunValCheck','off');
%modelFun = @(x,freq) LorentzianModel(x,freq);
for ii=1:size(data,2)
init(3)=freq_zf(ind_zf(ii));
    outputs_zf(ii,:) = nlinfit(freq_zf, real(spectra_zf(:,ii)).' , @(x,freq) LorentzianModel(x,freq), init, nlinopts); % add
weights to the data
end
%For this linewidth the amplitude is 199.3435; noiseless fitting CV is 0.1%
%limited by modeling options nlinopts
Error_zf=(amplitudes.'-outputs_zf(:,1)/199.3435*2)./amplitudes.';
CV_model_error_zf4=std(Error_zf)
%set the zero-filling factor
ZF=8;
% Fourier transform the time-domain data with zero-filling factor ZF
spectra_zf = fftshift(fft(data, 1024*ZF,1),1);
%Model the real part of the spectrum as an Absorption-mode Lorentzian
%signal (should be the exact model ignoring noise)
freq_zf=linspace(-500,500,1024*ZF);
%starting values (chosen without great care, but ballpark)
init=[200 -1 0]; %I suspect we will have to pick the offset starting value to be closer.
%Quick scan of the spectrum to find the max
[peaks ind_zf]=max(real(spectra_zf),[],1);
%starting values (chosen without great care, but ballpark)
outputs = zeros([n_offsets 3]);
nlinopts = statset('nlinfit');
nlinopts = statset(nlinopts,'MaxIter',400,'TolX',1e-6,'TolFun',1e-6,'FunValCheck','off');
%modelFun = @(x,freq) LorentzianModel(x,freq);
for ii=1:size(data,2)
init(3)=freq_zf(ind_zf(ii));
    outputs_zf(ii,:) = nlinfit(freq_zf, real(spectra_zf(:,ii)).' , @(x,freq) LorentzianModel(x,freq), init, nlinopts); % add
weights to the data
end
%For this linewidth the amplitude is 199.3435; noiseless fitting CV is 0.1%
%limited by modeling options nlinopts
Error_zf=(amplitudes.'-outputs_zf(:,1)/199.3435*2)./amplitudes.';
CV_model_error_zf8=std(Error_zf)
subplot(1,3,3)
plot([0 1 2 3],[CV_model_error_nozf CV_model_error_zf2 CV_model_error_zf4 CV_model_error_zf8 ],'o');
set(gca,'XTick',[0 1 2 3]);
set(gca,'XTickLabel',{'none','2','4','8'});
set(gca,'YLim',[0 0.09]);
```

bioRxiv preprint doi: https://doi.org/10.1101/2022.05.31.494144; this version posted June 1, 2022. The copyright holder for this preprint (which was not certified by peer review) is the author/funder, who has granted bioRxiv a license to display the preprint in perpetuity. It is made available under aCC-BY 4.0 International license.

```
%Include the Lorentzian Model used for fitting
function F = LorentzianModel(x,freq)

%F = ones(size(freq))./(x(2)^2*(freq-x(3)).*(freq-x(3))+1)*x(1)+x(4)*(freq-x(3))+x(5);
%Simplify the fuller model to keep things simple. Parameters are size,
%width and offset.
F = ones(size(freq))./(x(2)^2*(freq-x(3)).*(freq-x(3))+1)*x(1);

end
```
